# Molecular docking based screening of Noggin inhibitors

**DOI:** 10.6026/97320630014015

**Published:** 2018-01-31

**Authors:** Sindhura Gudipati, Ravi Muttineni, Archana Uday Mankad, Himanshu Aniruddha Pandya, Yogesh Trilokinath Jasrai

**Affiliations:** 1 Department of Botany, Bioinformatics and Climate Change Impacts Management, School of Sciences, Gujarat University, Ahmedabad 300019, India.; 2 Schrodinger, Bangalore 560086, India.

**Keywords:** NOG, small molecules, docking, BMP antagonist

## Abstract

Noggin (NOG) a BMP (bone morphogenetic protein) antagonist plays a key role in preferentially driving a subset of breast cancer cells towards the bone and causing osteolytic lesions leading to severe pain and discomfort in the patients. Owing to its role in bone metastasis, NOG could be promising molecular target in bone metastasis and that identifying small molecule inhibitors could aid in the treatment. Towards identifying cognate inhibitors of NOG, structure based virtual screen was employed. A total of 8.5 million ligands from e-molecule database were screened at a novel binding site on NOG identified by the Sitemap tool, employing GLIDE algorithm. Potential eight molecules were selected based on the Glide score, binding mode and H-bond interactions. Free energy of binding was calculated using Molecular mechanics based MMGBSA and the obtained energy was used in the prioritizing the compounds with the similar structures and glide score. Further, the compounds were evaluated for their druggability employing physico-chemical property analysis. Our study helped in identifying novel potential NOG inhibitors that can further be validated using in-vivo and in-vitro studies and these molecules can also be employed as tool compounds to study the functions of BMP.

## Background

Early diagnosis of breast cancer is pivotal in the maximizing the survival rates of the cancer patients. Often, breast cancers are detected only after they are metastasized. One of the major metastatic sites of the breast cancer is the bone [1]. Bone metastasis leads to pathological fractures, life threatening hypercalcemia, spinal cord compression, severe pain and morbidity. Understanding, the underlying molecular mechanisms in bone metastasis helps in identifying plausible novel targets, which could ameliorate pain and reduce morbidity.

Bone tissue is made up of osteoblasts, osteoclasts and osteocytes. Osteoblasts are involved in the bone formation, while osteoclasts in the re-sorption of the bone. RANKL (Receptor activated NF kappaBLigand) is a member of the tumor necrosis factor cytokine family and is responsible for osteoclast differentiation and activation. OPG (Osteoprotegrin) is an osteoblast-secreted decoy receptor that functions as a negative regulator of bone resorption. Always equilibrium is maintained between the RANKL and OPG. Shift of this equilibrium towards the RANKL results in lesions that destruct the bone conversely, shift towards OPG results in bone formation, which is brought about by the family of growth factors called Bone morphogenetic proteins (BMP) [2, 3]. Tumor cells release growth factors that stimulate osteoblasts to release RANKL that binds to the RANK (Receptor activated NF kappaB) present on the premature osteoclasts converting them to mature osteoclasts. Increased osteoclasts activity results in the osteolytic lesions characterized by the fractures and bone pain. BMP upregulates OPG through the activation of intra cellular messengers like SMADs (Mothers Against Decapentaplegic Homolog) which transcriptionally regulates RUNX2 (Runt Related Transcription Factor 2) [4]. Since physiological functions of BMPs are critical for bone formation, they are tightly regulated by a family of BMP antagonists that include Cerberus (Cer1), Twisted gastrulation (Twsg1), Chordin (Chrd), Crossveinless 2 (CV2) and Noggin (NOG) [5].

NOG is a secreted glycosylated homodimer and acts by directly binding to the BMP and preventing BMPs from binding to their receptors. NOG is preferentially expressed in the breast cancer cells that metastatize to the bone. It is involved in the numerous developmental processes. Binding of NOG to BMPs shifts the equilibrium between the RANKL and OPL towards RANKL there by resulting inosteolytic lesions [6].

Recent evidence suggests that NOG plays a significant role in the tumor growth and progression. Keratin 14-driven NOG over expression in mice results in development of skin tumors [7]. The osteolytic lesions in bones xenografted with the PC3 (human prostate cancer cell line) cells showed increased osteoclast activity and reduced osteoblast activity. Interestingly, when NOG-silenced PC3 cells were used repair activity was seen in lesions emphasizing the role of NOG in prostate cancer [8]. Expression of NOG in breast cancer cells provides them with bone colonization capabilities and also increased osteoclast activity and when NOG was silenced the osteoclast activity was reduced [9]. From these results we hypothesized that NOG inhibition could help in reducing bone metastatic cancer progression thereby alleviating pain in the metastatic bone lesions.

Previous studies by Karen et al. identified flavonoids that activate the BMP signaling pathway by inhibiting NOG [10]. Here for the first time we intended to identify small molecule inhibitors of NOG using structure based virtual screening that would possibly increase the available BMP levels, thereby may aid in restoring the bone damage and thus inhibit bone metastatic cancer progression. Alternatively, some of these molecules can be used, as tool compounds that would help to further understand the functions of NOG and BMPs in the context of various cancers. In order to achieve the above-mentioned objective we employed high throughput SBVS of small molecules.

## Methodology

### Protein preparation

Structure of the NOG was retrieved from PDB with the identification number 1M4U [11]. Loops missing in the PDB structure were modeled using SwissModel (https://swissmodel.expasy.org) [12]. To ensure correct starting structures initial structure of the protein was refined and subjected to energy minimization. The 3D model of the protein was prepared using the Protein Preparation Wizard in Maestro [13]. Protein was prepared by adding the hydrogen atoms, optimizing hydrogen bonds, removing atomic clashes, adding formal charges to the hetero groups and then optimizing at neutral pH. Finally the structure was minimized using optimized potential for liquid simulations (OPLS-2005) force field.

### Binding site analysis

Binding site was generated using the SiteMap tool from schrodinger [14]. SiteMap characterizes sites through a combination of properties calculated at each site point which include size of the site as measured by number of site points, the degree of enclosure by protein, degree of exposure to solvent, tightness with which sitepoints interact with the protein, hydrophobic and hydrophilic character of the site as well as the balance between these terms and degree to which a ligand and has possibility of donating or accepting hydrogen bonds. An overall SiteScore and druggability score are then used in selection of potential binding sites.

### Ligand and Library preparation

The Ligand and molecules from e-molecule database are retrieved in structure data file (SDF) format. The molecules were subjected to ligand preparation using Ligand Prep module of Schrodinger suite (Schrodinger). The ligand preparation process of molecules involves preserving the definite chiralities, to generate minimum five low-energy stereoisomers per ligand, using default conditions at pH 7.0 ± 2.0. The resulting ligands are subjected to high throughput virtual screening using the GLIDE (Grid based Ligand and Docking with Energetics) module of Schrodinger suite.

### Docking

Receptor grid was generated using receptor grid generation in the Glide application (Glide, version) of Maestro. Once receptor grid was generated, ligands were docked to the protein-using Glide docking protocol. The ligands were docked by using a three tire docking which starts with "High throughput Virtual Screening" (HTVS) followed by "Standard Precision" (SP) and then by "Extraprecision" mode (XP). The docked conformers were evaluated using Glide (G) Score [15, 16].

### MMGBA

The docked complexes were subjected to Molecular Mechanics/Generalized Born Surface Area (MM-GBSA) analysis for predicting the binding energy by prime approach. The binding energies, obtained through MM-GBSA OPLS-2005 are considered much more accurate than the XP GScore [17].

### ADME property analysis

ADME properties of selected ligands were analyzed using QikProp tool of Schrodinger suite. The tool predicts physiochemical properties with a detailed analysis of: (i) Molecular Weight (ii) partition coefficient (iv) hydrogen bond donors (v) hydrogen bond acceptors (vi) number of rotatable bonds

## Results

### Protein preparation and binding site analysis

NOG small molecule antagonist could be used locally to promote bone formation. In order to identify suitable binding pocket for virtual screening of small molecule inhibitors; binding site analysis was performed using SiteMap tool of Schrodinger software. SiteMap identified five sites based on site score that includes size, volume, amino acid exposure, enclosure, contact, hydrophobicity, hydrophilicity and donor/acceptor ratio. The site having site score and druggability score of 0.95 and 0.98 respectively was used for further screening of the novel compounds using docking protocol. The sites with site score of 1 and above can be a suitable site for the ligand binding. In addition druggability score of >1 is considered suitable for modulating the activity of protein with a small molecule [18]. The selected site having scores close to one is believed to have suitable binding site for the drug like compounds. The amino acids in proposed binding active site region are Leu 41, Pro 42, Leu 43, Val 44, Asp 45, Leu 46, Ile 47, Glu 48, Leu 164, Phe 168, Val 175, Gly 176, Ser 177, Cys 178, Ser 180, Lys 193, Pro 194, Arg 204, Ile 220, Try 222, Pro 223, Ile 224, and Ile 225.

### Small molecule Database preparation

The eMolecule database containing 7303475 compounds was used and filtered using Rapid Elimination Of Swill (REOS) which is a hybrid method that combines a set of functional group filters with some simple counting schemes with more than 200 rules [19]. A total of 5744923 compounds were obtained after REOS filtering and subsequently passed through the Pan-Assay Interference Compounds (PAINS) to remove non-specific compounds [20]. This filter further reduced the number to 5734778 compounds and these were clustered using molprint2D finger print with 64 bit and with Leader-follower clustering method using Canvas tool of Schrodinger software to obtain diverse compounds. Finally compounds were prepared using Ligand Preparation tool with pH 7 .0 (±1.0). Ligand preparation generated 8537456 compounds (maegz format) with proper ionization and tautomeric states were generated and are used for virtual screening.

### Virtual Screening and Identification of small molecule inhibitors

To identify small molecule inhibitors we have performed SBVS using prepared 8.5 million druggable compounds of eMolecule database against the selected site. Before running virtual screening a grid was generated on protein binding site using selected sitemap site to calculate potentials of binding site that are used for docking and scoring the compounds. The docking based screening was performed multi-tiered screening protocol, starting with HTVS followed by SP and XP methods [16]. The High Throughput Virtual Screening (HTVS) mode is designed to screen large libraries quickly with rough scoring functions, hence to screen 8.5 million compounds we started with this method. The top ranked hits (top 20%) were passed through Standard Precision (SP) mode, which is ten times slower and more precise than HTVS. The SP method is more exhaustive in conformational sampling and more precise than HTVS method with the expense of time. About 20,000 compounds obtained from SP method (best 50% of the compounds) were further evaluated with even more precise and more computationally intensive Extra Precision (XP) method. About 1000 compounds obtained from XP method were shortlisted based on docking score that are -9.0 and above. The high glide score indicated a high binding affinity towards the target. We checked for the following interactions, hydrogen bonds, salt bridges, halogen bonds, aromatic bonds, pi-cation and also pi-pi interactions all of which contribute towards the stability of the protein-ligand complexes. All the compounds formed NH-O with the backbone carbonyl of Gly 176. Except ligand 8 all the compounds formed NH-O with backbone atoms of Pro223. Except ligands 3, 4, 5 all the ligands interacted with back bone carbonyl atoms of the Ile 225 forming NH-O bond. Ligand 4 showed hydrogen bonds NH-O with the back bone carbonyl group of Asp 45. In the Ligand 6 sulfonyl oxygen forms NH-O bond with the backbone amino group of Asp 45. Ligand Ligand 7 forms OH-OH with the Asp 45 and Gln221. Carboxy group Ligand 8 forms the O-H bond with the backbone amino group of Leu 46. Ligand1, Ligand 4 and Ligand 5 form CH-O bond with the back bone carbonyl group of Pro223. Ligand 3 forms CH-O bonds with the side chain carbonyl group of Gln 221. The interactions are shown in the Figure 2.

### Molecular Mechanics Genralized Born Surface Area (MMGBSA)

**Figure 1:**
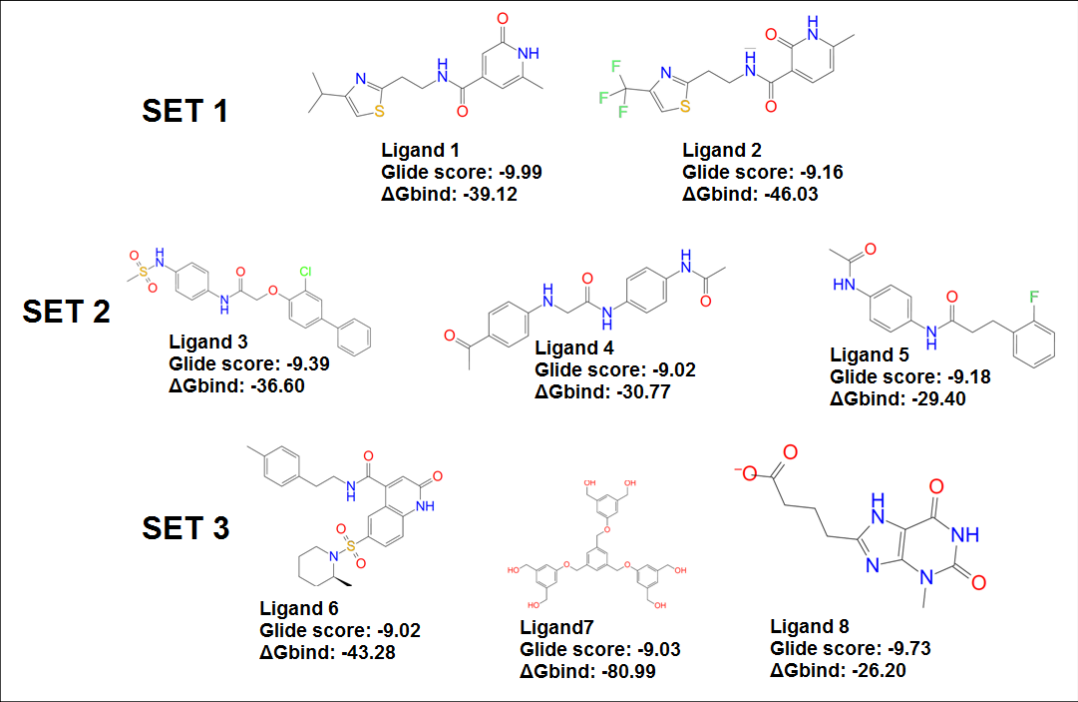
2D Structures of the compounds selected with corresponding Glide score and ΔGbind (kcal/mol).

**Figure 2:**
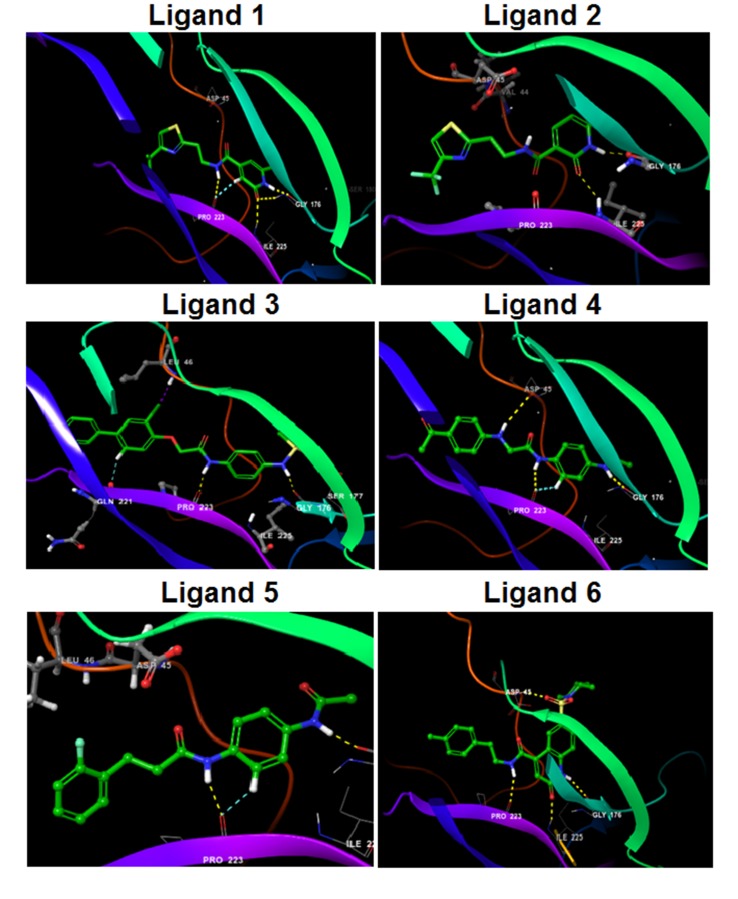
Docking pose of selected ligands of top six molecules and their amino acid interactions in the identified active site.Ligands are represented in ball and stick model and all the carbon atoms are colored in green, nitrogens in blue, oxygens in red, sulphur in yellow, fluorine in light green and chlorine in dark green. All H-bond interactions are represented in yellow dotted lines; CH-O interactions are represented as blue dotted lines and halogen interactions in purple dotted lines.

The ΔG bind between NOG and each ligand, respectively, were calculated using the MMGBSA and are shown in Figure 1. Ligand 7 showed the maximum ΔG bind and the ligand 8 showed the minimum ΔG bind.

### ADMET Analysis

In order to understand whether these compounds could further be utilized as drug molecules we performed ADMET analysis on the calculated ADMET properties. The analysis shows that all the selected molecules follow Lipinski's rule of five and number of rotatable bonds <10 with an exception of ligand 7, molecular weight and other parameters do not follow Lipinski's rule. Similarly ligand 8 is also predicted to have low oral absorption. The results are shown in Table 1.

## Discussion

Based on the similarity of the structure, molecules were grouped into five sets. Ligand 1 and Ligand 2 in set 1 has a substituted thiazole attached to ethylamide linker, which in turn is connected to pyridine ring. Amide -NH in the ligand 1 forms a NH-O interaction with backbone carbonyl atom of Pro 223. The pyridone carbonyl on ligand 1 and 2 interact with the backbone - NH of Ile 225 forming a NH-O interaction. Amine group in the pyridone on the ligand 1 and 2 forms NH-O bond with the backbone carbonyl atom of the Gly 176. Additionally, pyridone carbonyl on ligand 1 also forms a NH-O with the Gly176 resulting in a bifurcated H-bond. Since, each strong H-bond accounts to about 15 kcal of enthalpic stabilization bifurcated Hbond interactions are expected to contribute more stabilization in comparison with a single H-bond interaction. Ligand 1 also forms CH-O interaction with the backbone carbonyl atom of Pro 223. Next calculating the ΔG bind values helps in the prioritizing the potential compounds. Ligand 2 with a ΔG bind value of - 46kcal/mol and ligand 1 with a ΔG bind value of -39 kcal/mol is throwing a different picture in contrary to the earlier mentioned contribution of bifurcated H-bonds in ligand1. In this context, if molecules are short-listed just based on docking scores without binding energy might show a different order of priority in comparison when we judge them with binding free energy contribution. Therefore we prioritized ligand 2 in set 1 vis-a-vis ligand 1 from the same set. Set 2 consists of ligand 3, 4 and 5 with similar linker and small changes in the linker substitution attached to the sulphonamide in ligand 3 and acetamide group in ligands 4 and 5 respectively. In all the three ligands the amine group of the linker region forms a NH-O interaction with the backbone carbonyl group of Pro 223. The amine group in the sulphonamide of the ligand 3 and acetamide groups of ligands 4 and 5 forms a NH-O interaction with the backbone carbonyl of the Gly 176. Chlorine in ligand 3 forms Cl-HN interaction with the backbone -NH of the Leu 46 while secondary amine attached to phenylacetamide on the ligand 4 forms a bond with the NH-O of the Asp 45. Ligand 4 and 5 form CH-O interactions with the backbone carbonyl atoms of Pro 223, ligand 3 forms CH-O interaction with the side chain carbonyl of Gln 221. Glide score of the ligands 3, 4 and 5 is -9.39, -9.02, and -9.18 respectively. While binding free energies are -36.603, -30.770 and -29.401 kcal/mol. Based on the glide score and the binding energy ligand 3 is considered as a potent NOG inhibitor in the set 2 while ligand 4 and 5 have approximately same glide score and binding energy. Set 3 consisted of the ligands 6, 7 and 8. -NHof the linker forms NH-O interaction with the backbone carbonyl group of Pro 223, carbonyl oxygen forms NH-O hydrogen bond with the backbone carbonyl of Ile 225 and -NH of the benzo pyrridone ring formed NH-O interaction with the backbone carbonyl group of Gly176. The suphonyl oxygen forms hydrogen bond with the backbone amine group of Asp 45. A good glide score and the ΔG bind of -9.02 and -43.280 kcal/mol respectively and also abides to the Lipinski rule of 5, indicating that it could be a potential drug candidate. The ligand 7 in the set 4 had good glide score of -9.038 and binding energy of -80.995 kcal /mol, shows strong hydrogen bond interactions but owing to its poor physicochemical properties cannot be considered as a potential drug candidate. Ligand 8 although had a good glide score of -9.73 and binding energy was very high -26.20kcal /mol. Therefore ligand 8 hence is not a good candidate. In the light of the docking scores, hydrogen bond, binding energy and physicochemical properties we conclude that ligand 2, ligand 6 and ligand 1 to be potential Noggin inhibitors.

## Conclusion

In summary, we used structure based virtual screening to identify novel small molecule inhibitors of NOG. We first ranked the compounds based on their glide score and binding mode. The compounds with the glide score greater than -9.0 were further analyzed for the hydrogen bond interactions. Our analysis indicated that the all the ligands interacted with the backbone carbonyl oxygen of the Gly 176 of β2 region. Pro223 and Ile225 are the key residues involved in the binding. Further, we utilized binding free energy for the selection of potential inhibitors. ADMET analysis was used to understand the physic chemical properties and drug likeliness of the compounds. Three compounds, Ligand 2, Ligand 6 and ligand 1 are found to be potential NOG inhibitors that had a good glide score, binding energy and had favorable physiochemical properties so as to be considered as drugs. These molecules can be used in the prevention and as adjuvant therapy in bone metastasis. Additionally, these can also be used, as tool compounds to further understand the function of the NOG and BMP.

## Figures and Tables

**Table 1:** QikProp results of selected compounds based on docking score. a) Molecular weight of the molecule b) Predicted octanol-water partition coefficient (log Po/w) (–2.0 to 6.5) c) Number of rotatable bonds < 10 d) number of hydrogen bond donors ≤5 e) number of hydrogen bonds acceptors ≤5 f) Percentage human oral absorption (% ABS) (>80% is high, <25% is poor).

Molecule	mol_MW	QPlogPo/w	No of rotatable bonds	DonorHB	Acceptor HB	%Human Oral Absorption
a	b	c	d	e	f
Ligand 1	305.394	2.4	5	2	6.5	90.661
Ligand 2	331.312	3.378	4	0	4.5	96.68
Ligand 3	430.905	3.582	7	2	7.75	91.706
Ligand 4	325.366	1.805	6	3	8	79.402
Ligand 5	300.332	3.326	5	2	5	100
Ligand 6	467.582	3.306	6	2	9.5	88.215
Ligand 7	576.642	2.96	21	6	12.45	35.379
Ligand 8	252.229	-0.013	4	3	7	43.528
